# Low level of dark personality traits in transgender people and their relationships with resilience

**DOI:** 10.3389/fpsyt.2025.1674117

**Published:** 2025-11-07

**Authors:** Agnieszka Mateja, Barbara Gawda

**Affiliations:** 1 Institute of Psychology, Humanitas University, Sosnowiec, Poland; 2 Prof. Department of Psychology of Emotion and Personality, Maria Curie-Sklodowska University, Lublin, Poland

**Keywords:** transgender, dark personality tetrad, resilience, narcissism, Machiavellianism, psychopathy, sadism

## Abstract

**Introduction:**

In addition to anxiety disorders and depressive symptoms, transgender people are also shown to have pathological personality profiles. These patterns are due to functioning under chronic stress, exposure to discrimination, victimization, the inability to affirm gender identity, and insufficient social support. The internalized transphobia predisposes transgender individuals to psychological decompensation. The study aims to assess Dark Personality Trait among transgender individuals and to establish the relationships between Dark Tetrad traits and resilience.

**Materials and Methods:**

The Dark Tetrad (narcissism, psychopathy, Machiavellianism, sadism) was assessed using The Short Dark Tetrad Scale (SD4-PL). Resilience was measured using The Resilience Measurement Scale (SPP-25) questionnaire. The dimensions of psychological resilience were also evaluated, including perseverance, determination in action, openness to new experiences, sense of humor, personal competence in coping, tolerance of negative emotions, tolerance for failure, viewing life as a challenge, optimism, and the ability to mobilize in difficult situations. In the statistical analysis, a Multivariate Analysis of Covariance (MANCOVA) was conducted. Correlations between dark personality traits in the transgender and cisgender groups were compared using Fisher’s z-test.

**Results:**

The study results indicate a slightly lower level of narcissism and Machiavellianism in transgender women compared with cisgender women, and a slightly increased level of sadism in all men, regardless of whether they are transgender or cisgender. No differences were observed between the transgender and cisgender groups in terms of dark personality traits. Transgender individuals exhibited significantly lower level of general resilience than cisgender individuals.

**Conclusions:**

The results of participants from the transgender group indicate lower level of dark personality traits. Observed differences in dark personality traits are related to gender and are independent of transgenderism. Psychological resilience provides a subtle protective function against the development of dark personality traits.

## 1 Introduction

Transgender individuals, as a sexual minority, experience discrimination and social prejudice, which leads to increased levels of stress that seriously affect their mental health, as well as causes psychological problems such as depressive and anxiety disorders (1–3).

Since the onset of identifying as transgender can usually be traced to adolescence or early adulthood, the personality traits that develop dynamically during this period may evolve as dysfunctional coping mechanisms. For example, transgender individuals may cope with severe gender dysphoria through social isolation, avoidance of close relationships, self-harm, outbursts of uncontrolled anger, or envy of the less problematic lives of others (4–9).

Over time, negative experiences of discrimination, harassment, and rejection by close ones generate the belief that transgender identity is impossible to accept and is invalidated. Chronic invalidation leads to self-invalidation, disrupting the process of self-acceptance (10–13).

Some researchers believe that transgender patients may present with various mental disorders (14), while others suggest that transgender identity is associated with personality disorders (15). In international studies, different personality scales have been used to understand the personality and psychological traits of transgender individuals. The results are inconsistent, which may be due to the small sample sizes (14–17).

Transgender identity is associated with internal, familial, occupational, social, financial, and health-related crises. The internal tension associated with the crises may occur on several levels. The first involves internal recognition of gender incongruence. Current societal norms, which assume cisgender identity as the default, necessitate the denial of cisgender identity in the process of recognizing one’s transgender identity. For this reason, the process of self-awareness regarding psychosexual identity in transgender individuals is often delayed (18, 19). The next problem generating stress and crisis is the act of coming out as transgender. Another stress-inducing process is undergoing gender transition (20). Health issues are primarily mental, although hormone therapy and various procedures undertaken to conceal external sexual characteristics that conflict with one’s experienced gender often carry adverse effects on physical health (21, 22).

There is some data in the literature, albeit not very extensive, pointing to pathological traits and/or personality profiles in transgender individuals (4, 23–26). The cross-sectional studies have evaluated prevalence estimates of personality pathology in the transgender population (17, 27, 28). Cluster B PDs and particularly borderline and narcissistic PDs, were identified as the most frequently diagnosed Axis II disorder in transgender people (29). Almost half of the included transgender clients exhibited at least one PD diagnosis (4). Therefore, we assume that differences between transgender and cisgender individuals may manifest in what are called dark personality traits, as there are gender differences in these traits. Dark personality traits include: Machiavellianism, sadism, psychopathy, and narcissism (30–35).

Machiavellianism is characterized by a focus on self-interest and the desire to achieve personal goals regardless of circumstances and at any cost. Numerous studies indicate that men score higher on Machiavellianism than women (36). In women, Machiavellianism is correlated with harm-avoidance, anxiety, and sensitivity or hypersensitivity. In men, it is associated with risk-taking, self-confidence, and an opportunistic worldview. Sex differences in Machiavellianism are explained by a theoretical perspective grounded in evolutionary theory. According to this view, sex differences in traits and values result from innate predispositions that developed in response to ancestral adaptive demands (37). Individuals can more freely express their internal tendencies, including those related to gender, if they have a sense of security provided by resource availability (36–38). By contrast, the socio-cultural perspective assumes that with greater gender equality, men are forced to compete not only with other men but also with women. This process is reflected in increases in Machiavellianism among men. At the same time, women derive greater benefits from increased gender equality and therefore need not rely to the same extent on Machiavellian strategies (39). In societies with greater gender equality, individuals tend to compare themselves across genders more frequently (36, 40). At the individual level, the apparent increase in the gender gap in Machiavellianism is driven by women’s reactions: women increasingly disapprove of Machiavellian tendencies in themselves, and consequently, Machiavellianism decreases among women, while remaining stable among men (36). The increase in resources available to women in countries with greater gender equality does not force women to compete for those resources. Consequently, women are not compelled to adopt Machiavellian strategies to achieve their goals. Women may more freely display characteristics associated with femininity that conflict with Machiavellian principles.

Another personality trait that manifests differently depending on the gender is narcissism. Differences in narcissism can be explained, among other factors, by reference to social roles. The level of narcissism is lower in women (41). Various behaviors observed in women are attributed to internal trait dispositions, which are then internalized as gender-typical traits, to which individuals compare their own behavior (3, 42). Narcissism involves agentic characteristics such as dominance and self-confidence; therefore, according to social role theory, this explains the higher level of narcissism in men (43–46). Men tend to exhibit lower levels of agreeableness, which is an antagonistic trait to narcissism (37, 41, 47). Women more often than men assume roles and work in occupations that promote high agreeableness, for example, nursing, teaching, and childcare. Gender-dependent socialization experiences may influence the level of narcissism and its antagonistic trait, agreeableness (48).

Sadism, in turn, has been the least extensively studied compared to other dark personality traits. It appears to be conditioned by environmental factors such as avoidant attachment patterns, exposure to domestic violence, and negative parenting styles (49, 50). This trait seems to function as an adaptation to the conditions transgender individuals live in. In this sense, it serves as a way to ensure dominance, protection, and control in hostile, violent environments (50, 51). From an evolutionary perspective, these traits, commonly perceived as negative, may be interpreted as adaptive responses to hostile, violent environments. In such environments, survival and competition for resources require the development of specific behavioral strategies. Adverse childhood experiences, which transgender individuals often encounter during this developmental stage, constitute critical events that influence the development of personality traits, including dark personality traits (23, 52–55). Psychological aggression is likely the strongest predictor of tendencies toward behaviors generally defined as sadism, and more precisely, behaviors based on exerting control over others. Aggressive behaviors experienced are internalized as a way of establishing dominance.

Justification of the role of psychological resources as resilience in our study on the personality traits of transgender individuals is of great importance, as personality pathology may affect the clinical symptoms of gender dysphoria (56). A precise understanding of the personality functioning of transgender individuals, determination of identity stability, self-regulation, and interpersonal relationships are important prognostic indicators that may help identify deficits and resources that should be considered in the transition process. Mental health problems are a significant, sometimes primary and at other times secondary, source of stress and may complicate the process of exploring gender identity and coping with gender dysphoria (57, 58). For example, symptoms of dysfunctional personality traits may lead to reduced adherence to medical and therapeutic recommendations, which require meticulous and regular engagement, while simultaneously disturbing the already challenging transition process.

Clinical consultations with transgender individuals should focus on psychological development, with particular attention to personality development. This study aims to enhance understanding of transgender individuals, mitigate discrimination and prejudice, and underscore the importance of building a supportive environment. Furthermore, the results may help create personalized treatment and psychological support plans for transgender individuals, thereby helping them cope with difficulties and improving their mental health and quality of life.

In a comprehensive analysis of health processes, we use knowledge of human resources and deficits as well as their environment. The context of environmental influences and individual traits in specific situations may support or hinder the process of meeting needs, fulfilling life tasks, and achieving goals. Resources and risk factors constitute the context conditioning the adaptation process (59–63). Key resources include psychological resilience, which conditions adaptive coping with stress experienced by transgender individuals. In our study, we decided to focus on resilience, considered an important individual resource, as well as personality traits that may represent a risk factor hindering the adaptation process, necessary during gender transition. Of particular importance for transgender individuals appears to be the general level of psychological resilience and its two aspects, perseverance and determination in action, and an optimistic outlook on life, with the ability to mobilize in difficult situations. At the same time, we chose not to control the level of minority stress in our study, as numerous reports confirm the presence of high levels of minority stress among transgender individuals (24, 64–67).

## 2 The current study

Taking into account the above-described findings presenting transgender people as characterized by pathological personality traits, first, we aim to assess the potential differences between transgender and cisgender individuals in terms of both dark personality traits. Second, we aim to identify the relationships between psychological resilience and dark personality traits such as sadism, psychopathy, narcissism, and Machiavellianism representing the Dark Personality Tetrad, in transgender men and women. We hypothesize that there are differences between transgender and cisgender people and that gender differences can play an important role in this differentiation. We consider resilience as an important covariant, as resilience can be a protective factor during the development of dark personality traits.

## 3 Materials and methods

### 3.1 Participants

The study involved 242 participants. The sample was divided into two groups, clinical and control. The clinical group comprised 104 transgender respondents, while the control group included 138 cisgender individuals. Cisgender identity was determined based on anonymous self-identification by the participants. The control group was selected for the study according to gender. The control group was selected at random. The gender imbalance in the group of transgender individuals (77% men, 23% women) results from the predominance of transgender men in the general transgender population and the lack of access to transgender women. The problem of gender imbalance in studies of transgender individuals is a common issue in research on this group. This may distort study results, and we should be aware of this imbalance (68). However, in pour study the clinical group and control group were matched in term of gender. The age of transgender participants ranged from 18 to 52 years (M = 25.32; SD = 7.65). The age of cisgender participants ranged from 18 to 58 years (M = 34.35; SD = 10.13).

Sociodemographic data information collected through the survey are presented in **Table 1**.

**Table 1:** Sociodemographic data information collected through the survey.

Survey data	Transgender group		Cisgender group	
	Numbers of persons	Percentage (%)	Numbers of persons	Percentage (%)
Education				
Primary	0	0	1	.72
Middle school	0	0	2	1.45
Vocational	13	12.50	5	3.62
Secondary	72	69.23	55	39.85
Bachelor’s degree	0	0	20	14.49
Master’s degree	19	18.27	55	39.86
Place of residence				
Village	14	13.46	22	15.94
Small town	6	5.77	19	13.77
Medium- sized city	38	36.54	32	23.19
Large city	46	44.23	65	47.10
Marital status				
Single	72	69.23	78	56.52
Married	0	0	45	32.61
Divorced	0	0	14	10.14
Widowed	0	0	1	.72
Informal relationship	32	30.77	0	0

### 3.2 Procedure

Recruitment of individuals for the clinical group was conducted among patients undergoing psychological evaluation as part of the gender transition process at the Rehabilitation and Medical Center Fizjomedica in Tychy, Poland. All transgender patients had been referred to the clinic by a sexologist overseeing their transition process, which allows the assumption that their transgender identity had been professionally confirmed. The group of transgender individuals is a clinical group, as it included persons undergoing the gender transition process and therefore subjected, among others, to psychological, psychiatric, endocrine, and neuroimaging assessments. The inclusion criteria were as follows: meeting the ICD-10 diagnostic criteria for “transsexualism”, being at least 18 years of age, participation in the gender transition process before legal gender change, voluntary participation in the study. The exclusion criterion was a self-declared non-binary identity (14 individuals were excluded from the study on this basis). The proportions of women and men in both groups are the same.

Participation in the study was voluntary and anonymous. Participants were informed of this both prior to the study and through a notice included at the beginning of each questionnaire. The study was conducted individually with each participant. Completing the questionnaire, which included socio-demographic data and psychometric instruments, took approximately 15 minutes.

The study was submitted to and received a favorable recommendation from the Research Ethics Committee of the Humanitas University (Poland, Sosnowiec; Opinion no. 4/2025).

### 3.3 Measures

The study assessed variables comprising the Dark Personality Tetrad (narcissism, psychopathy, Machiavellianism, sadism), as well as the overall level of psychological resilience and its specific components.

Measurements were obtained using the following psychological instruments:

The Resilience Measurement Scale (SPP-25) (69) and the Short Dark Tetrad Scale (SD4) (70). Both questionnaires have demonstrated strong psychometric properties. An original survey questionnaire was also used to characterize the clinical and control groups. Within this survey, participants responded to questions regarding gender, age, education, place of residence, marital status, presence of illness, and medication use.

The SD4 measures antisocial personality traits, including Machiavellianism, narcissism, psychopathy, and sadism (70). SD4-PL is the Polish adaptation of the Short Dark Tetrad (SD4) questionnaire developed by D.L. Paulhus, E.E. Buckels, P.D. Trapnell, and D.N. Jones. The Polish version was adapted by P. Debski, P. Palczynski, M. Garczarczyk, M. Piankowska, K. Haratyk, and M. Meisner (71). The Polish version of SD4 is currently undergoing psychometric testing. The SD4-PL version was obtained from the authors of the study. The SD4-PL scale consists of 28 statements rated on a 5-point Likert scale, where 1 indicates Strongly disagree and 5 indicates Strongly agree. It is a newly developed scale, methodologically constructed during the work on the adaptation article (71, 72). Cronbach’s alpha coefficients in the validation study: psychopathy is.71, narcissism is.79, Machiavellianism is.68, sadism is 77. Cronbach’s alpha coefficient of the entire scale in the current study is.68.

The SPP-25 was developed by Zygfryd Juczynski and Nina Oginska-Bulik (69). It demonstrates good psychometric properties. Cronbach’s alpha is 0.89, the standard error of measurement for the overall score is 3.81, and the reliability of individual subscales ranges from.67 to.75, which is considered satisfactory. The data comes from the validation study. Cronbach’s alpha coefficient in the current study is as follows.92. The SPP-25 scale consists of 25 statements rated on a 5-point Likert scale, where 0 indicates Definitely Not and 4 indicates Definitely Yes. The scale measures aspects of psychological resilience understood as personality traits. It can be used to assess personality-based predispositions in individuals, particularly those exposed to stress. The tool is based on self-report. The SPP-25 provides a general resilience score as well as levels of the following five resilience components:

1. Perseverance and determination in action.
2. Openness to new experiences and sense of humor.
3. Personal competence in coping and tolerance of negative emotions.
4. Tolerance of failure and perceiving life as a challenge.
5. Optimistic outlook on life and the ability to mobilize oneself in difficult situations (69).

### 3.4 Data analysis plan

The descriptive statistics are presented in **Table 2**. Skewness and kurtosis values indicate that the distributions of the variables do not deviate from the normal distribution. To account for more variables, a two-way (2 groups x 2 genders) Multivariate Analysis of Covariance (MANCOVA) was applied. Dependent variables were narcissism, psychopathy, Machiavellianism, and sadism. The covariate in our study was resilience. Then, a similar two-way MANCOVA was conducted with five components of resilience as covariates. Next, correlations between the dark tetrad personality traits and resilience were calculated separately for both groups, i.e., transgender and cisgender, and these correlation values were compared between the two groups using a Fisher’s z test.

**Table 2:** Descriptive statistics (n=242).

Variables	Min.	Max.	M	SD	Skewness		Kurtosis	
					Statistics	SE	Statistics	SE
Resilience	22.00	100.00	66.65	16.59	-.321	.156	-.659	.312
Res1	3	20	13.53	3.90	-.373	.156	-.877	.312
Res2	2	20	15.18	3.178	-.937	.156	.998	.312
Res3	3	20	12.90	3.95	-.339	.156	-.738	.312
Res4	5	20	13.61	3.58	-.647	.156	-.218	.312
Res5	1	20	11.44	4.43	-.212	.156	-.386	.312
Machiavellianism	5	35	21.19	4.84	-.302	.156	.672	.312
Narcissism	7	35	18.61	5.60	.23	.156	.046	.312
Psychopathy	7	33	14.15	4.85	.984	.156	1.119	.312
Sadism	7	33	17.17	6.63	.180	.156	-.788	.312

Res1- Perseverance and determination in action, Res2- Openness to new experiences and sense of humor, Res3- Personal competence in coping and tolerance of negative emotions, Res4- Tolerance of failure and perceiving life as a challenge, Res5- Optimistic outlook on life and the ability to mobilize oneself in difficult situations, SE- standard error.

The data from this study are not publicly available due to the sensitive nature of the studied population.

## 4 Results

A two-way MANCOVA was conducted to examine the combined effects of group (transgender vs. cisgender) and gender (women vs. men), while controlling psychological resilience as total score on the Dark Tetrad.

Multivariate tests revealed significant effects for group (Wilks’ λ = .75; *F*(4, 234) = 19.96; *p* <.001; η_p_^2^ = .25), gender (Wilks’ λ = .88; *F*(4, 234) = 8.24; *p* <.001; η_p_^2^ = .12) and their interaction (Wilks’ λ = .93; *F*(4, 234) = 4.33; *p* = .002; η_p_^2^ = .07).

**Table 3** presents the results of univariate tests for between-subject effects, showing that group membership differentiated levels of Machiavellianism, and sadism. Gender differentiated Machiavellianism, psychopathy, and sadism. The interaction between group and gender was significant for Machiavellianism and narcissism. No statistically significant differences were found for the remaining Dark Triad dimensions in relation to the analyzed factors or their interaction (**Table 3**).

**Table 3:** Summary of univariate tests for between-subject effects for the Dark Tetrad explanatory model.

Effect		*F*	*p*	η_p_^2^
Psychological Resilience	Machiavellianism	.38	.541	
	Narcissism	39.08	<.001	.14
	Psychopathy	5.90	.016	.02
	Sadism	7.00	.009	.03
Group	Machiavellianism	3.98	.047	.02
	Narcissism	.68	.412	
	Psychopathy	1.86	.174	
	Sadism	24.00	<.001	.09
Gender	Machiavellianism	12.67	<.001	.05
	Narcissism	1.10	.294	
	Psychopathy	14.56	<.001	.06
	Sadism	20.36	<.001	.08
Group * Gender	Machiavellianism	4.86	.028	.02
	Narcissism	4.60	.033	.02
	Psychopathy	.20	.654	
	Sadism	.29	.592	

The covariates in the model were estimated as follows: Resilience = 66.65.

A detailed analysis of the comparison of mean scores showed that transgender individuals scored significantly higher on sadism (Δ*M* = -5.02; *p* <.001) than cisgender individuals. Women scored significantly lower on Machiavellianism (Δ*M* = -2.54; *p* <.001), psychopathy (Δ*M* = -2.71; *p* <.001), and sadism (Δ*M* = -3.72; *p* <.001) compared to man (**Table 4**).

**Table 4:** Mean scores of the dark personality traits by group and gender.

Dark Tetrad	Group	Gender	*N*	*M*	*SD*
Machiavellianism	transgender	Women	27	17.85	3.94
		Men	77	22.05	3.16
		Total	104	20.96	3.83
	cisgender	Women	106	21.13	5.61
		Men	32	22.13	5.07
		Total	138	21.36	5.49
Narcissism	transgender	Women	27	17.52	5.37
		Men	77	17.65	4.64
		Total	104	17.62	4.82
	cisgender	Women	106	18.74	5.86
		Men	32	21.41	6.28
		Total	138	19.36	6.04
Psychopathy	transgender	Women	27	13.15	2.30
		Men	77	15.19	5.10
		Total	104	14.66	4.62
	cisgender	Women	106	13.08	4.66
		Men	32	16.03	5.47
		Total	138	13.76	5.00
Sadism	transgender	Women	27	19.52	3.07
		Men	77	22.36	5.11
		Total	104	21.63	4.82
	cisgender	Women	106	13.80	6.03
		Men	32	17.88	6.13
		Total	138	14.75	6.28

M, mean; SD, standard deviation.

**Figure 1:**
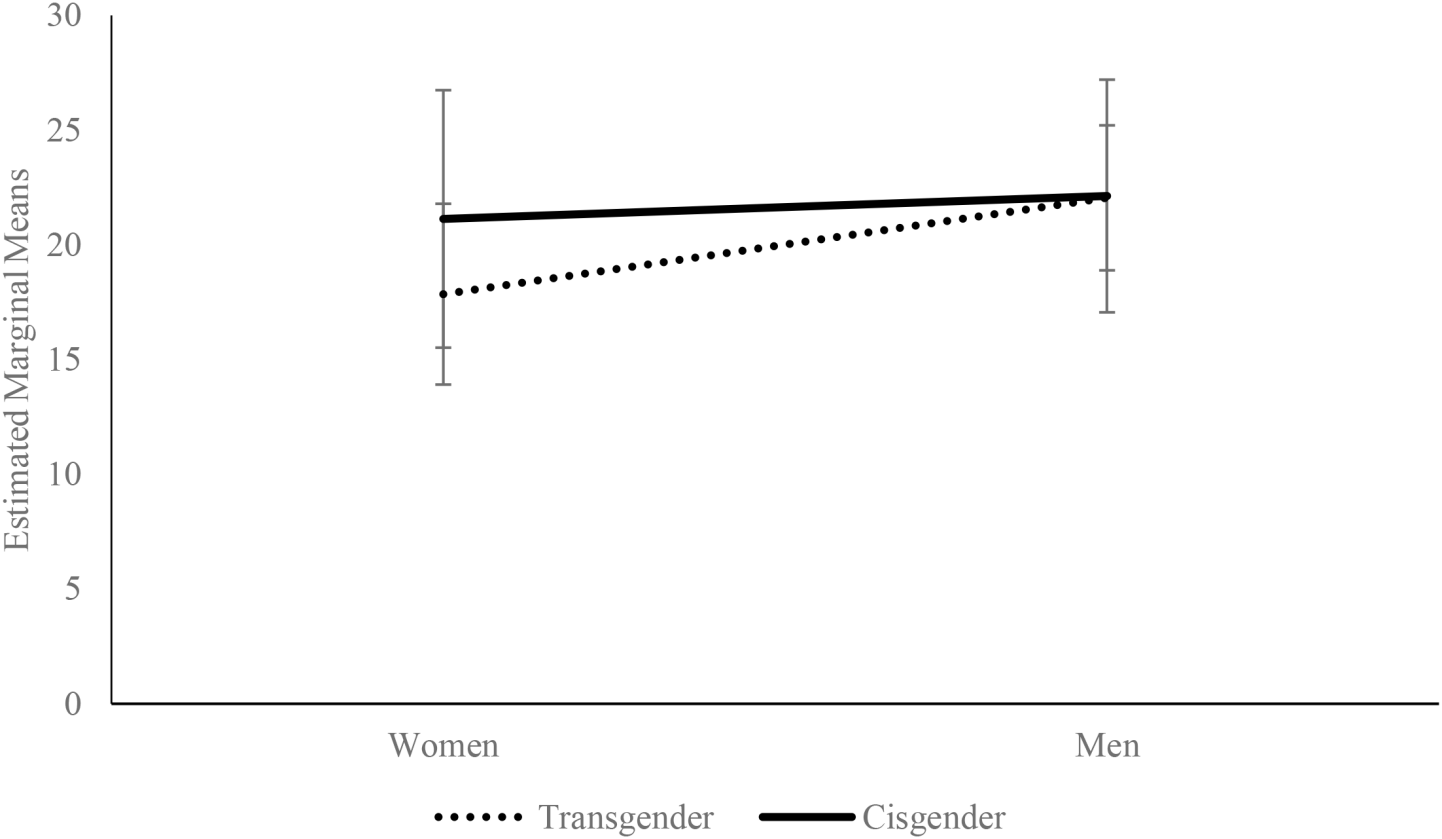
Estimated marginal means (± 1 SD) for Machiavellianism by gender and group. The covariates in the model were estimated as follows: Resilience = 66.65.

A simple effects analysis of group on Machiavellianism revealed that transgender women exhibited significantly lower levels of Machiavellianism than cisgender women Δ*M* = -3.08) (**Figure 1**). This effect was not significant in the male group F(1,237) = .003; *p* = .956; η_p_^2^ <.01).

Although both groups and gender differentiate sadism, there is no significant interaction effect of gender and group on sadism. In both groups, i.e., transgender and cisgender, sadism is higher among men (**Table 5**).

**Table 5:** Estimated marginal means (± 1 SD) for Machiavellianism, by gender and group.

Variable	Groups	Gender	M	SE
Machiavellianism	Transgender	Women	17.995	.936
		Men	22.108	.545
	Cisgender	Women	21.077	.466
		Men	22.052	.841
Sadism	Transgender	Women	18.803	1.081
		Men	22.086	.629
	Cisgender	Women	14.076	.538
		Men	18.239	.971

The covariates in the model were estimated as follows: Psychological resilience = 66.65.

**Figure 2:**
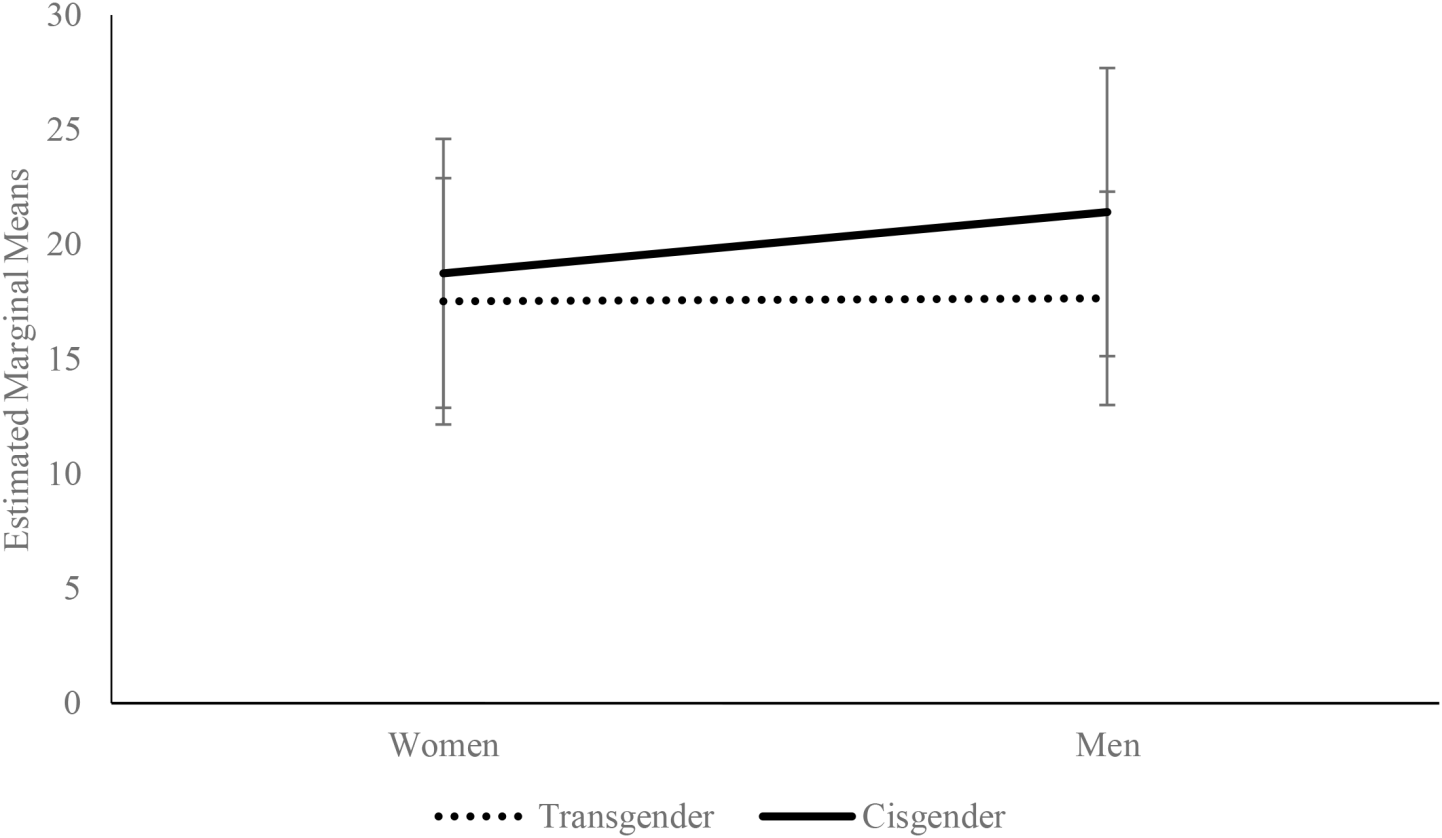
Estimated marginal means (± 1 SD) for Narcissism, by gender and group. The covariates in the model were estimated as follows: Resilience = 66.65.

A simple effects analysis for narcissism by group indicated that transgender men exhibited significantly lower levels of narcissism than cisgender men, *F*(1,237) = 4.50; *p* = .035; η_p_^2^ = .02; Δ*M* = -2.33. This effect was not significant in the female group, *F*(1,237) = 0.72; *p* = .398; η_p_^2^ = .003. Analysis of simple effects for gender showed that within the cisgender group, women exhibited significantly lower narcissism levels compared to men, *F*(1,237) = 5.74; *p* = .017; η_p_^2^ = .02; Δ*M* = -2.47, whereas no gender differences were observed among transgender individuals (**Figure 2**).

A second two-way MANCOVA (multivariate analysis of variance) was conducted. This analysis examined potential interactive effects of group (transgender vs. cisgender) and gender (women vs. men). Five covariates were controlled for: perseverance and determination, openness to new experiences and sense of humor, personal competence and tolerance of negative emotions, tolerance of setbacks and treating life as a challenge, and optimism, and the ability to mobilize oneself. The dependent variable was the Dark Tetrad.

**Figure 3:**
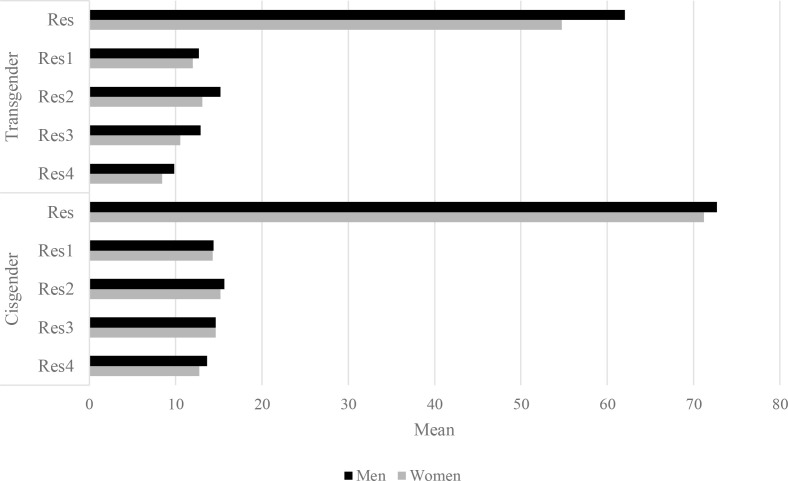
Mean levels of general resilience and resilience components (cisgender vs transgender participants) for dimensions with significant interaction effects. Res- Resilience, Res1- Perseverance and determination in action, Res2- Openness to new experiences and sense of humor, Res3- Tolerance of failure and perceiving life as a challenge, Res4- Optimistic outlook on life and the ability to mobilize oneself in difficult situations.

Multivariate tests revealed significant effects for psychological resilience components (Wilks’ λ = .72; *F*(4, 234) = 23.00; *p* <.001; η_p_^2^ = .28). Univariate analyses showed the significant effects of group and gender on dark personality dimensions while controlling for resilience components. Transgender individuals exhibited significantly lower levels of resilience than cisgender individuals (Δ*M* = -11.430). Multivariate tests revealed significant effects for group, as well as significant effects for four of the five covariates. Transgender individuals exhibited significantly lower levels of perseverance and determination in action than cisgender individuals (Wilks’ λ = .73; *F*(4, 234) = 20.84; *p* <.001; η_p_^2^ = .27; Δ*M* = -1.828). Transgender individuals display significantly lower levels of openness to new experiences and sense of humor than cisgender individuals (Wilks’ λ = .94; *F*(4, 234) = 3.73; *p* <.006; η_p_^2^ = .06; Δ*M* = -.935). Transgender individuals exhibited significantly lower levels of tolerance for failures and treating life as challenges than cisgender individuals (Wilks’ λ = .94; *F*(4, 234) = 3.86; *p* <.005; η_p_^2^ = .06; Δ*M* = -2.364). These individuals exhibited significantly lower levels of optimism and ability to mobilize in difficult situations than cisgender individuals (Wilks’ λ = .90; *F*(4, 234) = 6.58; *p* <.001; η_p_^2^ = .26; Δ*M* = -3.50). The only statistical interaction effect of group and gender on Machiavellianism was found while controlling for resilience components. The effect is similar to that of including the overall resilience score as a covariate. To better illustrate the results obtained, **Figure 3** displays the average levels of the general resilience score and its components, for which significant interaction effects were identified.

Subsequently, correlations between psychological resilience and dark personality traits were calculated (**Table 6**). Pearson’s correlation coefficient was used, as skewness analysis confirmed a normal distribution in both groups. A significant correlation between psychological resilience and narcissism was found in both groups. The correlation is positive and moderate. The higher the resilience, the higher the narcissism. A weak negative correlation was found between resilience and psychopathy in transgender group. It indicates that the higher the resilience, the lower the psychopathy. A similar weak negative correlation between resilience and sadism was found. The value of Fisher’s z-test indicates no significant difference in correlations between the transgender group and cisgender group (**Table 6**).

**Table 6:** Pearson’s correlations between resiliency and the Dark Tetrad in the transgender and cisgender groups.

Variable	Resilience		
	Transgender group	Cisgender group	Fisher’s z
Machiavellianism	.004	.118	-.87
Narcissism	.428**	.346**	.73
Psychopathy	-.196*	-.075	-.94
Sadism	-.086	-.171*	.66

*p<.01, **p<.05.

## 5 Discussion

Our study results do not confirm higher levels of dark personality traits in transgender people, although the literature on the subject suggests it. When comparing the two groups, researchers found differences in various personality traits (4). However, when we consider more variables, such as gender, the differences are found to be unrelated to transgenderism.

Our study results indicate a slightly lower level of narcissism and Machiavellianism in transgender women compared with cisgender women, and a slightly increased level of sadism in all men, regardless of whether they are transgender or cisgender. No differences were observed between the transgender and cisgender groups in terms of dark personality traits. Transgender individuals exhibited significantly lower levels of resilience than cisgender individuals for general resilience and four of the five of its components, such as perseverance and determination in action, openness to new experiences and sense of humor, tolerance for failures and treating life as challenges, and optimism and ability to mobilize in difficult situations.

The slightly lower level of Machiavellianism and narcissism in transgender women may be a consequence of hormonal treatment (73, 74). All cisgender women participating in the study underwent hormone therapy. Year by year, more transgender individuals, and at younger ages, begin hormone therapy. This is not due to an increase in the number of transgender people, but to reduced barriers to access to healthcare and to increased acceptance and destigmatization in society. The rise in the number of people undergoing hormone therapy concerns transgender women to a greater extent, because transgender women have historically experienced higher levels of stigmatization. Earlier initiation of hormone therapy may amplify characteristics typical of women, including lower levels of narcissism and Machiavellianism (75). Transgender women were subjected, during the first several, and in some cases many, years of life, to a masculine model of socialization and later internalized a feminine model. They may have displayed feminine traits earlier and been treated differently by their environment. Protective factors against anxiety symptoms in transgender women include self-esteem and good interpersonal functioning (76). The present study indicated that psychological resilience may mitigate the severity of dark personality traits, but the protective effect is subtle. We had assumed the protective effect would be stronger. This is an important correlation that permits the development of individualized therapeutic intervention plans for transgender women (77).

The gender gap is larger in countries with lower levels of gender equality. In Poland, gender inequality is greater than in Western European countries and the United States; therefore, Machiavellianism among the women in the studied sample is only slightly lower (78–80). In societies with greater gender equality, inter-gender comparisons are more salient and, consequently, gender identity and conformity to social gender norms are more clearly defined. This process may be somewhat stronger among transgender women, which is supported by the findings of the present study. Transgender women are perceived as less feminine and may perceive this as a threat to their femininity. This elicits reactive responses that increase differences between genders. This is manifested in the slightly lower level of Machiavellianism in transgender women compared with cisgender women. Thus, the mechanism that operates among women in societies with greater gender equality may be somewhat stronger in transgender women (36, 81–83). We do not observe an increase in the affirmation of male gender identity that might be expected to be threatened by gender equality, nor do we note an increase in Machiavellianism among transgender men relative to cisgender men. Gender equality does not reinforce a masculine pattern of gender identity (84–86).

The slight, uniformly elevated value of sadism in all men may be caused by men’s tendency toward competition and dominance. This could be connected to testosterone levels (14, 87). In conflictual situations or those perceived as difficult, men’s levels of tension rise and are expressed as anger, rage, and sometimes aggression. Thus, the slight increase in sadism among men may be related to their methods of regulating negative emotions. This issue, however, requires further research (51, 88, 89). It has been observed that the antagonism domain has been reported to show marked gender differences, with men scoring consistently higher than women (90). Manipulativeness, Grandiosity, Collousness, and Hostility, as facets included in the Antagonism domain, seem therefore to be less characteristic of the personality functioning of transgender men as opposed to cisgender men. One speculative explanation for this result could be ascribed to a lower propensity of transmen to a sense of entitlement and self-assertiveness, as they navigate a world in which they are more exposed to aggressions, microaggressions, harassment, and violence than cisgender men (91, 92).

Further research into the personality traits of transgender individuals is needed. Understanding the psychological functioning of transgender people will allow therapeutic processes to be adapted accordingly and reduce the level of minority stress (93). As a result, the quality of life of transgender people will improve.

### 4.1 Limitations

The interpretation of results regarding personality traits among transgender individuals is limited by the lack of comparable studies and the insufficient volume of such measurements. Additionally, participants in these studies are often at various stages of transition, which significantly affects psychological functioning (94).

Transgender research populations are often limited to those receiving clinical care. This study aimed to avoid that bias by recruiting individuals seeking the mandatory psychological evaluation required for transition, not necessarily those being treated for psychiatric disorders.

A significant challenge lies in assembling a gender-diverse research group. As in many studies, this one showed a predominance of transgender men (74%) compared to transgender women (26%). Efforts were made to minimize the impact of this imbalance by deliberately selecting a gender-balanced control group.

Another issue is the self-measure techniques we used. Self-report measures usually provide a less accurate description of personality functioning than the clinician assessment.

## 5 Conclusions

The study results indicate a slightly lower level of narcissism and Machiavellianism in transgender women compared with cisgender women, and a slightly increased level of sadism in all men, regardless of whether they are transgender or cisgender. No differences were observed between the transgender and cisgender groups in terms of dark personality traits. Transgender individuals exhibited significantly lower levels of resilience than cisgender individuals for general resilience and four of the five components, such as perseverance and determination in action, openness to new experiences and sense of humor, tolerance for failures and treating life as challenges, and optimism and ability to mobilize in difficult situations.

## Data Availability

The original contributions presented in the study are included in the article/supplementary material. Further inquiries can be directed to the corresponding author.
